# Merensky-type platinum deposits and a reappraisal of magma chamber paradigms

**DOI:** 10.1038/s41598-019-45288-8

**Published:** 2019-06-19

**Authors:** Sofya Chistyakova, Rais Latypov, Emma J. Hunt, Stephen Barnes

**Affiliations:** 1 School of Geosciences, University of the Witwatersrand, Johannesburg, South Africa; 2 CSIRO Mineral Resources, Kensington, Perth, WA 6151 Australia

**Keywords:** Petrology, Geochemistry, Economic geology, Mineralogy

## Abstract

Most of the world’s economically-viable platinum deposits occur as ‘reefs’ in layered intrusions – thin layers of silicate rocks that contain sulphides enriched in noble metals. There are two contrasting magmatic hypotheses for their formation. The first suggests accumulation through gravity-induced settling of crystals onto the magma chamber floor. The alternative argues for *in situ* crystallization, *i*.*e*. upward growth from the floor. Here we report on our discovery of the Merensky Reef in the Bushveld Complex that occurs on subvertical to overturned margins of depressions in a temporary chamber floor. Such relationships preclude crystal settling and demonstrate that the reef crystallized *in situ*. This finding indicates that platinum deposits can grow directly at the chamber floor, with immiscible sulfide droplets sequestering ore-forming noble metals from strongly convecting silicate magmas. Our model also provides evidence for the paradigm that argues for magma chambers being masses of nearly crystal-free melt, which gradually loses heat and crystallizes from the margins inward.

## Introduction

**Figure 1:**
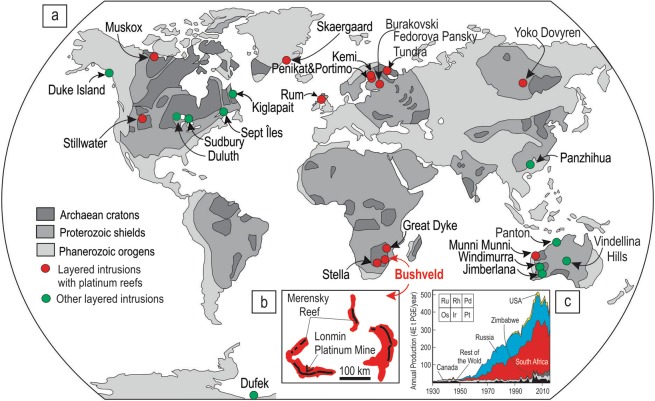
Distribution of layered intrusions worldwide, a contour of the world’s largest layered intrusion - the Bushveld Complex in South Africa and global PGE mine supply. (**a**) The world sketch map showing the locations of some well-known layered intrusions hosting the Merensky-type platinum deposits. Some other layered intrusions are also indicated. Map is slightly modified from the ref.^6^. (**b**) Schematic contour of the Bushveld Complex indicating the location of the Merensky Reef (black solid line). Also shown is the position of the Lonmin Platinum Mine, a major study area of this paper. (**c**) A diagram showing that more than half of global PGE mine supply is sourced from the platinum deposits of the Bushveld Complex in South Africa. Diagram is slightly modified from ref.^7^.

Merensky-type platinum deposits in mafic-ultramafic layered intrusions (Fig. 1a) – named after the classical Merensky Reef (MR) of the Bushveld Complex in South Africa (Fig. 1b) – are key repositories of strategically important metals (Fig. 1c), which are essential for the sustainable development of modern human society and a major petrological challenge for almost a century^1–7^. The Merensky-type platinum deposits occur as thin, continuous layers of sulphide- and chromite-bearing silicate rocks anomalously enriched in the platinum group elements (PGE; Os, Ir, Ru, Rh, Pt and Pd), thus are referred to as ‘platinum reefs’. The physical and chemical processes responsible for their origin remain highly controversial, with two opposing magmatic models. One school of thought holds that they are produced by gravity-induced settling of crystals through the magma body onto the chamber floor^8–16^, with potential subsequent sorting of minerals during late-stage slumping of the cumulate pile^17,18^. The competing hypothesis states that they are formed by *in situ* growth of all minerals, including chromite and sulphides, directly on the chamber floor, accompanied by minor re-deposition of the minerals in association with convection in the magma chamber^19–27^. The resolution of this dilemma remains critical for our understanding of the formation of magmatic mineral deposits as well as for critical evaluation of the competing paradigms for magma chamber evolution^28–52^.

**Figure 2:**
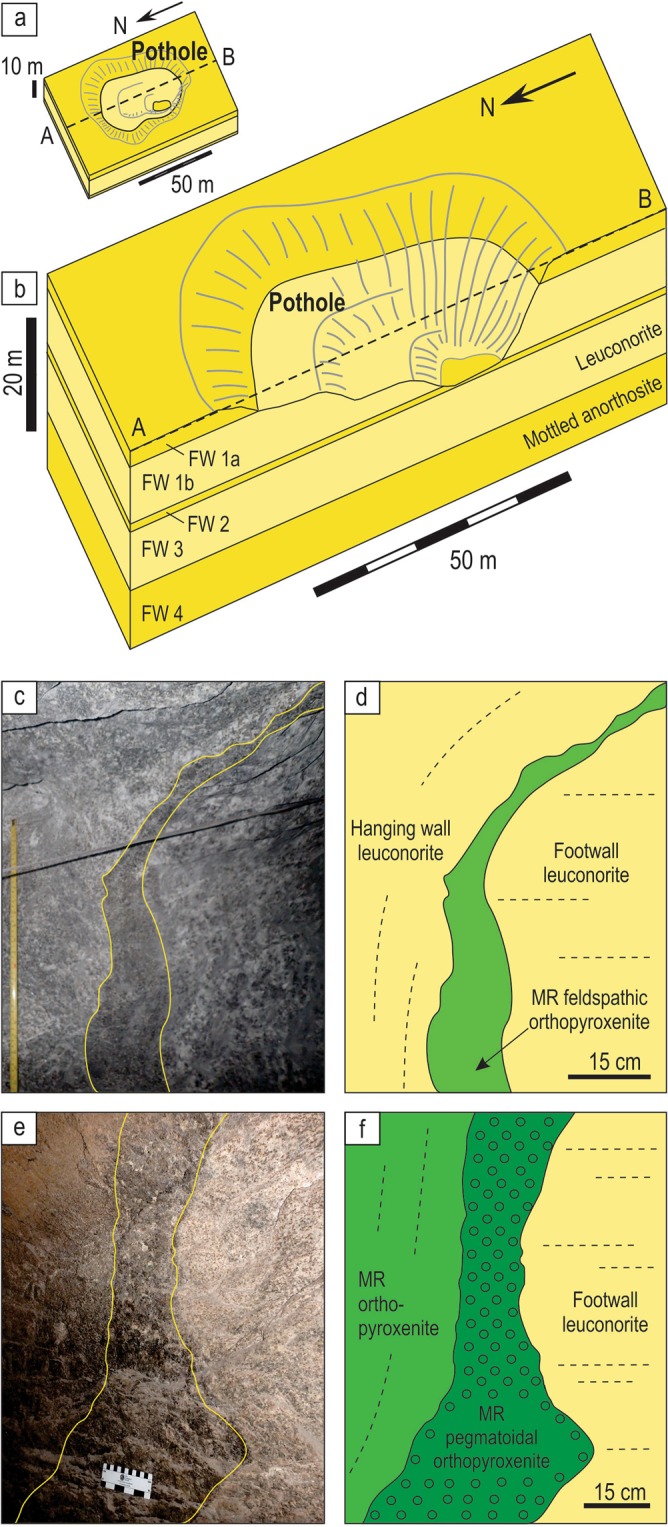
Schematic diagrams depicting typical morphology of a pothole associated with the MR of the Bushveld Complex and photos and sketches of underground exposures showing development of the MR package along subvertical to overhanging margins of potholes. (**a**) Schematic plan view of a typical pothole. (**b**) 3D model showing plan and cross section views through a typical pothole. FW – footwall rocks, HW – hanging wall rocks. (**c**,**d**) MR package is composed of pegmatoidal feldspathic orthopyroxenite and normal orthopyroxenite; the footwall is leuconorite 25 mW 21 RSE; (**e**,**f**) MR package is composed of feldspathic orthopyroxenite sandwiched between footwall and hanging wall leuconorite, 22 mE 23 RSE. Dashed lines indicate igneous layering in cumulates. All exposures are from Shaft 3 of the Karee mine, Lonmin Platinum, Western Bushveld Complex. Location of the mine in the Western Bushveld is shown in Fig. 1b.

We have approached the problem of Merensky-type platinum deposits by re-examining field relationships of the MR in the Bushveld Complex (Fig. 1b). This complex is the largest mafic magmatic body in the Earth’s continental crust^17,30^ which partly explains why the MR, together with two other principal ore deposits of the complex (UG2 chromitite and the Platreef), provide ~75% of the globally exploited platinum, ~35% of the world’s Pd and >80% of its Rh^2^ (Fig. 1c). Our choice is mostly governed by the fact that much of what we know about the platinum deposits has been historically derived from the intense study of this classic location. The MR usually occurs as a package of sulphide-bearing silicate rocks of broadly melanoritic to orthopyroxenitic composition (a few cm to a few m in thickness) that are extremely enriched in the PGE. The package is normally easily recognizable both in the field and laboratory because it occurs among cumulate rocks (anorthositic to pyroxenitic in composition) that are almost totally devoid of chromite, sulphide and PGE^14,17,24,25^. A remarkable feature of the MR is potholes – circular to elliptical depressions several metres to hundreds of metres in diameter^24,25,53–57^ (Fig. 2a,b). The potholes cut into the underlying cumulates to depths of up to several tens of metres and show margins that change from gently to steeply inclined and rarely become subvertical to locally overturned. Development of potholes with such a highly irregular surface topology is most commonly attributed to disruption and subsequent thermochemical erosion of the floor cumulates by new pulses of hot/superheated replenishing magmas^24–26,53,56,57^.

Here we present new field, textural and geochemical observations from the Lonmin Platinum Mine in the Western Bushveld (Fig. 1b), together with some literature data, that offer a remarkably simple solution to the challenge of platinum-reef formation. Our principal finding is that, in potholes, the entire MR package may locally develop along steeply dipping portions of potholes and even the undersides of overhanging sidewalls. This evidence is inconsistent with the hypothesis that the MR formed by crystal settling on the chamber floor – an idea that is deeply entrenched in explanations of platinum reefs in layered intrusions^1–5,8–16^ – and indicate, instead, that *in situ* growth of the MR on irregular surface of the chamber floor appears to be the only explanation for these geometries. The discovery thus provides the most impressive evidence for the concept that Merensky-type platinum deposits in layered intrusions do not require long-range settling of crystals; they can be easily formed from crystals growing *in situ*; that is, attached to the solidifying chamber margins. In the broader context, these new observations question the validity of gravity settling^28–39^ that has historically been the principal paradigm for explaining fractionation in evolving magma chambers^28–31^. Instead, the observations provide strong support for the concept that magma chambers develop from almost crystal-free liquids, which predominantly crystallize from the chamber floor upwards and inwards from the margins^40–52^. This also implies that the chemical evolution of melt in magma chambers occurs through physical separation of evolved liquids from crystals growing *in situ* by compositional convection^41,42,47,48,58^.

## Results

**Figure 3:**
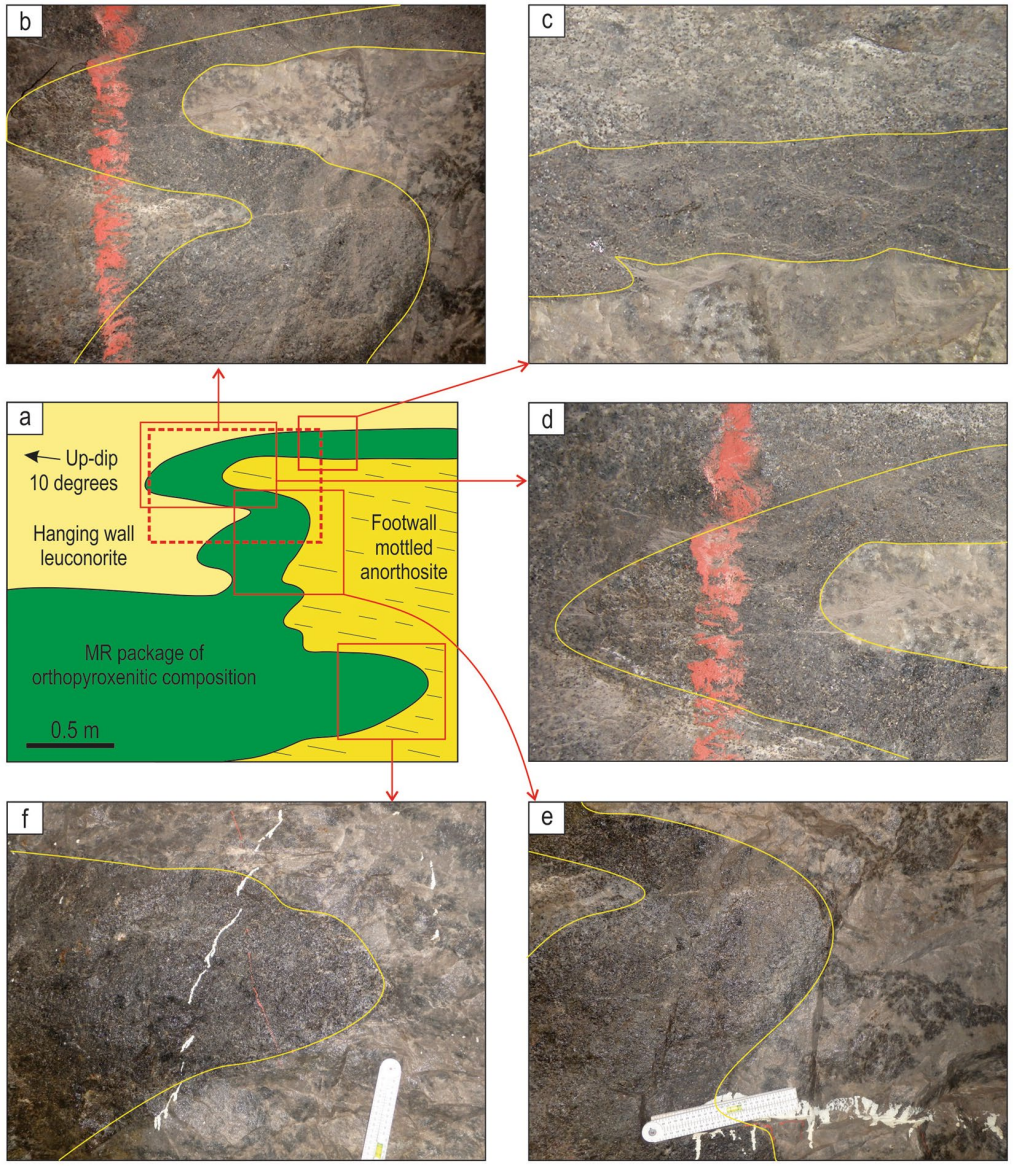
Sketch and close up photos of an underground exposure showing development of the MR package along subvertical to overhanging margins of a pothole in the Western Bushveld Complex. Sketch is based on visual identification of rocks in the field. The MR package is of broadly orthopyroxenitic composition and is sandwiched between footwall mottled anorthosites and hanging wall leuconorite. (**a**) Sketch of the exposure of a MR pothole sidewall; (**b**) MR package draping over a finger-like projection of footwall anorthosites; (**c**) MR package sitting on a planar shoulder of the pothole; (**d**) close-up of the MR package enveloping the top of a finger-like projection of footwall anorthosites; (**e**) MR package developing on the overhanging sidewall; (**f**) MR package undercutting footwall anorthosites. The exposure is from Shaft 3 of the Karee mine, Lonmin Platinum, Western Bushveld Complex, 25 mW 21 RSE.

Our field examination of potholes at the Lonmin Platinum Mine in the Western Bushveld (Fig. 1b) has revealed one fundamental, but hitherto overlooked feature: in some underground exposures the entire MR package drapes over all irregularities (domes and depressions) of the temporary chamber floor. The best examples show a MR package developed along steeply inclined, vertical and even overturned sidewalls of a pothole (Fig. 2c–f). The MR package comprises highly mineralized pegmatoidal to normal feldspathic orthopyroxenite and poorly-mineralized feldspathic orthopyroxenite to melanorite. These are hosted by almost barren, footwall and hanging norite/leuconorite to mottled anorthosite. Importantly, the discordant relationships of the MR package with igneous layering in the footwall leuconorite (Fig. 2c–f) indicate that the vertical to overhanging position of these rocks is primary and not related to any late-stage deformational processes (e.g. slumping). We have studied one of these exposures in detail (Fig. 3). It represents one side of ~2 m deep pothole that occurs within the footwall mottled anorthosite (Fig. 3a). The highly irregular surface of the footwall anorthosite is consistently draped by a 20–50 cm thick package of the MR orthopyroxenite/melanorite that is well developed along sub-horizontal (Fig. 3c), steeply inclined, subvertical and overhanging sidewalls (Fig. 3b,d,e). At the base of the pothole, the MR package increases to over a meter in thickness and in one point cuts into the footwall and is wholly hosted in mottled anorthosite (Fig. 3f). No chromitite seams are associated with the MR package in this exposure. The footwall mottled anorthosite displays characteristic development of ortho- and clinopyroxene oikocrysts, some of which are truncated by the pothole margins (Fig. 3b–f). The MR package is overlain by hanging wall leuconorite (Fig. 3a,b).

**Figure 4:**
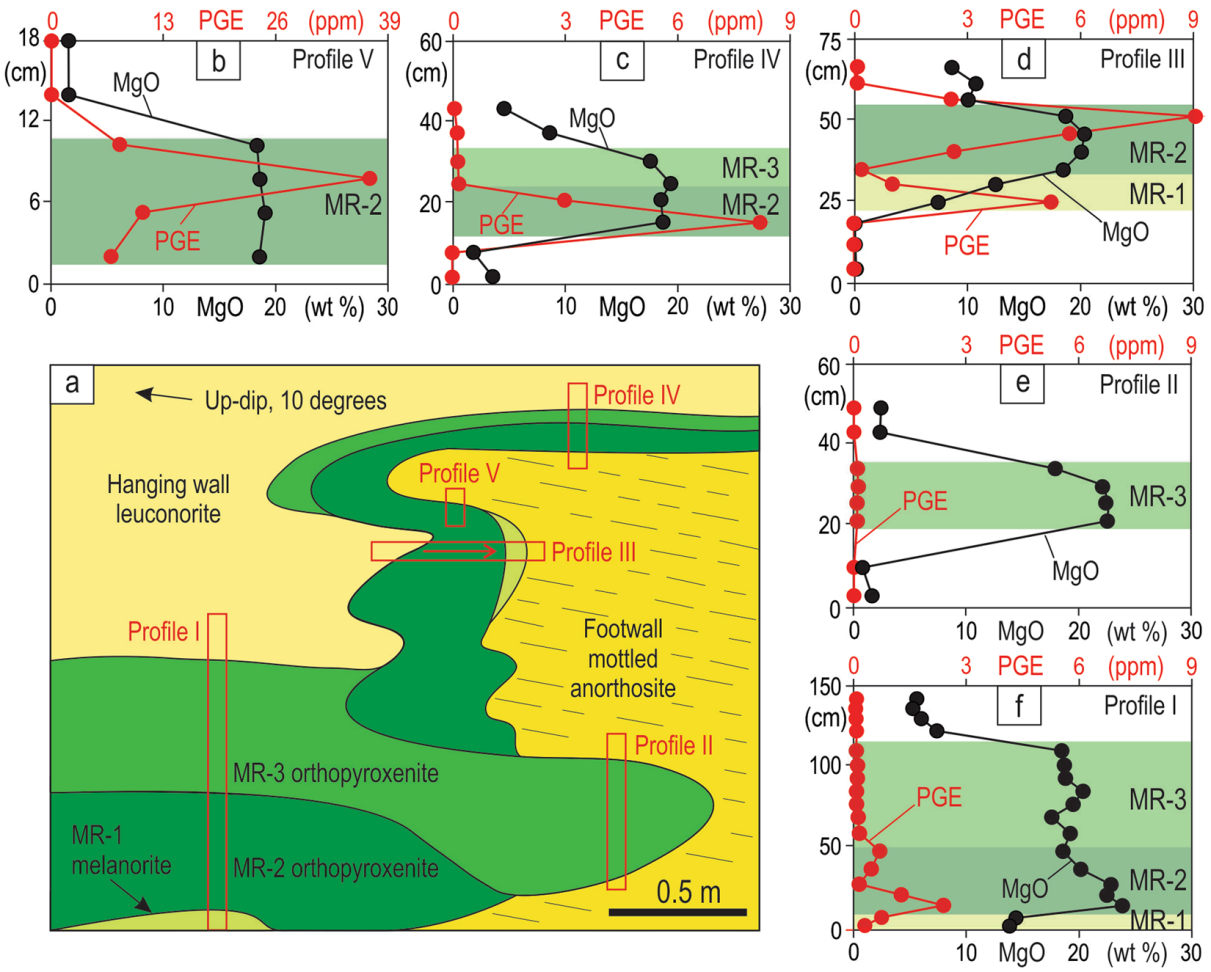
Sketch of the underground exposure from Fig. 4 showing positions of the five sampling profiles (I-V) across the MR package of the Western Bushveld Complex. The sketch is based on petrographical and chemical identification of rocks in the laboratory. (**a**) sketch of the exposure with interpreted MR sublayers; (**b**–**f**) show whole-rock MgO and PGE variations in profiles across the MR package. The chemistry reveals a complex internal structure of the MR package consisting of, at least, three sublayers - PGE-rich melanorite (MR-1), PGE-rich orthopyroxenites (MR-2) and PGE-poor orthopyroxenites (MR-3). The exposure is from Shaft 3 of the Karee mine, Lonmin Platinum, Western Bushveld Complex, 25 mW 21 RSE. Dashed line denotes layering defined by oikocrysts in the footwall mottled anorthosite. For illustrative purposes, the footwall anorthosites are shown as containing no PGE although they were not analysed for PGE. Data are presented in Supplementary Information Table 1.

**Figure 5:**
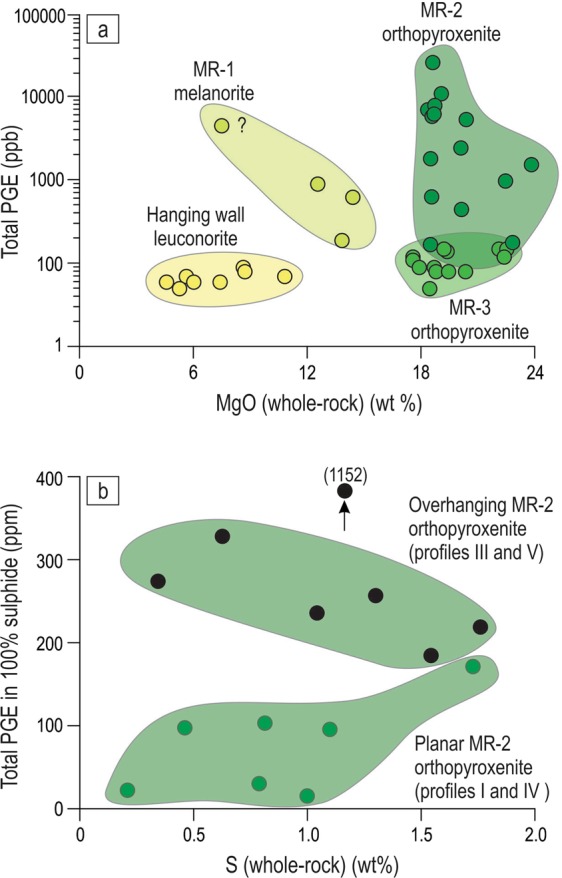
PGE abundances and tenors in sublayers of the MR package of the Western Bushveld Complex. (**a**) Binary plot of whole-rock MgO versus total PGE content illustrating differences between the three sublayers of the MR package and the hanging wall leuconorite. The data are from the five sampling profiles in Fig. 5. The footwall anorthosites are not included as they were not analysed for the PGE. (**b**) Binary plot of whole-rock S versus PGE tenors (total PGE content in 100% sulphide) showing that MR-2 orthopyroxenite on overhanging portions of the chamber floor (in pothole) has higher PGE tenors compared to that on the planar portions of the chamber floor. Location of profiles is shown in Fig. 5. Data are from the underground exposure in Shaft 3 of the Karee mine, Lonmin Platinum, 25 mW 21 RSE. Data are presented in Supplementary Information Table 1.

**Figure 6:**
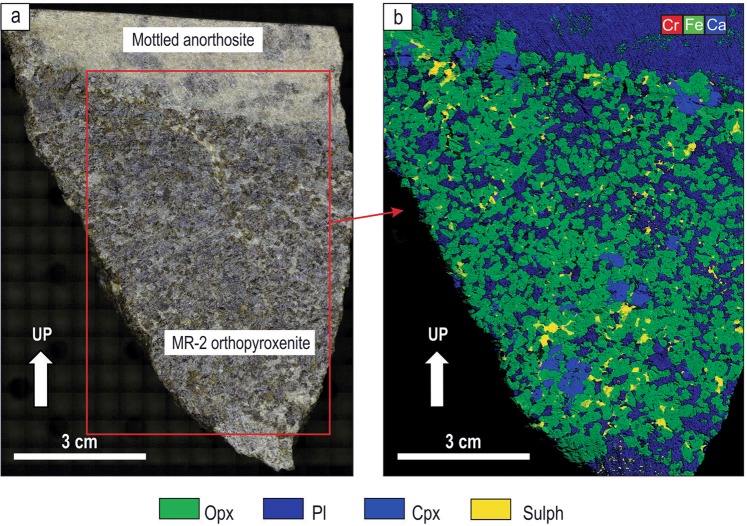
Photograph (**a**) and XRF image (**b**) of the uppermost portion of overhanging MR-2 orthopyroxenite that drapes over overhanging sidewalls of the pothole and contains disseminated sulphides (sampling profile V in Fig. 5). 25 mW 21 RSE, Shaft 3 of the Karee mine, Lonmin Platinum, Western Bushveld Complex. Opx, orthopyroxene; Pl, plagioclase; Cpx, clinopyroxene; Sulph, sulphide.

A diamond saw was used to sample the exposure at several structural positions (Fig. 4a, profiles I-V) to examine petrographical and chemical characteristics of the MR package. Detailed study reveals that the MR package is not homogeneous but instead consists of three sublayers with distinct petrographies and whole-rock chemistries, especially PGE abundances. The lowermost sublayer is composed of highly mineralized melanorite (MR-1; 13–15 wt% MgO), the middle consists of highly mineralized orthopyroxenite (MR-2; 19–24 wt% MgO) and is overlain by poorly mineralized orthopyroxenite (MR-3; 17–23 wt% MgO) (Fig. 4). The chemical differences between the MR sublayers and host rocks are evident from both the vertical profiles (Fig. 4) and a binary plot of MgO versus total PGE (Fig. 5a). Importantly, individual vertical profiles show varying combinations of the sublayers: i.e. profiles II and V (MR-3 and MR-2), profiles III and IV (MR-1, MR-2 and MR-2, MR-3) and profile I (MR-1, MR-2, MR-3). Chemical and petrographical observations from the profiles suggest that the sublayers are in transgressive relationships with the footwall, each other and the hanging wall. The relative sequence of rock formation appears to be footwall anorthosite, PGE-rich melanorite (MR-1), PGE-rich orthopyroxenite (MR-2), PGE-poor orthopyroxenite (MR-3) and finally hanging wall leuconorite. One interesting consequence of the transgressive relationships between the MR sublayers is that each of them can be locally in direct contact with the footwall rocks. It is worthwhile noting that the portion of the MR-2 sublayer that is richest in sulphides and PGE (up to 38 ppm PGE; profile V) does not occur in the planar portions (the base or shoulder) of the pothole, but is underneath the uppermost, finger-like projection of footwall anorthosites (Figs 4a,d, 6). Also importantly, the highest values of the PGE tenor (i.e. total PGE in 100% sulphide) are observed in MR-2 orthopyroxenite developed along subvertical to overhanging margins of a pothole (profiles III and V) rather than along the planar chamber floor (profiles I and IV) (Fig. 5b). Finally, the MR package is abruptly overlain by leuconorite with a substantially lower orthopyroxene content (5–10 wt% MgO).

**Figure 7:**
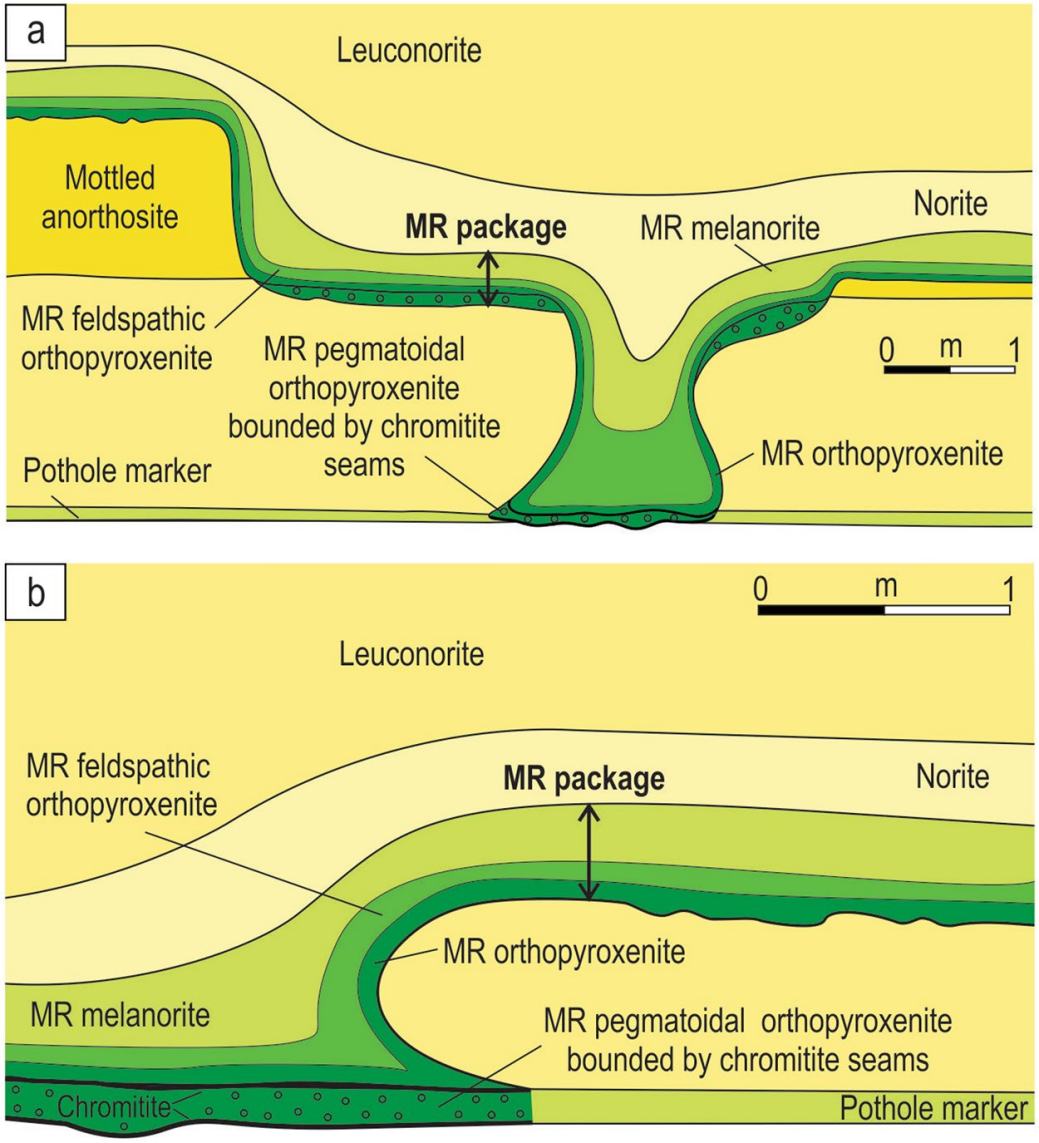
Sketches of underground exposures showing development of the MR package along subvertical to overhanging margins of potholes in the Bushveld Complex. (**a**) MR package that occurs on planar, subvertical and overhanging sidewalls of a pothole at the Waterfall West Shaft, Rustenburg Platinum Mine, Western Bushveld. (**b**) MR package that occurs on planar, subvertical and overhanging sidewalls of a pothole at the Townlands Shaft, Rustenburg Platinum Mine, Western Bushveld. Both sketches are slightly modified from ref.^59^.

Our subsequent literature search has revealed that overhanging MR packages, similar to ones presented here, have been documented earlier in potholes of the Western Bushveld^59^ (Fig. 7), but left unnoticed. The earlier reported MR packages are also composed of sulphide-bearing, normal to pegmatoidal feldspathic orthopyroxenites, which are sandwiched between footwall and hanging wall cumulates and develop along both the planar and subvertical to overhanging sidewalls of the potholes (Fig. 7). Again, the cross-cutting relationships of the MR packages with the footwall leuconorite (Fig. 7) suggest that the vertical to overturned position of these rocks is a primary magmatic feature. Thus, our new field, petrographical and chemical observations (Figs 2–4), together with the literature data (Fig. 7), collectively indicate that the development of the entire MR package on overhanging portions of the chamber floor is a very important petrological constraint. Until now, it has been only known that such style of development is characteristic of a thin basal chromitite seam (2–3 cm thick) of the MR^24,25^. The realization that this is true for the entire MR package drastically changes a situation and allows to employ this field-based constraint for much more rigorous testing of rivalling hypotheses for the origin of Merensky-type platinum deposits in layered intrusions.

## Discussion

Our discovery that the entire MR package (Figs 2–4, 7) develops as a ‘rind’ covering all the chamber floor depressions and culminations, even where these are vertical or overhanging; precludes the possibility that it forms by crystal settling at the chamber floor because sinking crystals cannot penetrate nearly solidified cumulate rocks forming overhangs. Clearly, such relationships can only be reconciled with the growth of silicate minerals and sulphides directly at the magma-cumulate interface. The *in situ* origin of the MR has been earlier proposed on the basis that its thin basal chromitite seam locally occurs along the overhanging margins of potholes^24,25^. The idea has been, however, questioned by ascribing this seam a non-magmatic origin. It has been interpreted as either metasomatic in origin, i.e. produced within unsolidified norites in response to their melting/dissolution by magmatic volatiles derived from the underlying cumulate pile^60^; or restitic in origin, i.e. formed by incongruent melting of orthopyroxene-rich footwall rocks by new hot magma pulses^61^. However, our finding that the entire MR package, even without any chromitite seams (Figs 2–4), still develops on the undersides of pothole sidewalls decisively undermines the above objections. If so, the MR of the Bushveld Complex – the canonical example of platinum reefs in layered intrusions – must represent an *in situ* crystallization phenomenon, with its high PGE status being attained while all its minerals, including sulfides, were heterogeneously nucleating and crystallizing against pre-existing minerals along the margins of the chamber. This is the most fundamental conclusion of our study that can probably be applied to platinum deposits in other layered intrusions as well as potentially extended to other types of magmatic deposits (e.g. chromite and Fe-Ti-V magnetite ores) in mafic-ultramafic complexes (Fig. 1a).

**Figure 8:**
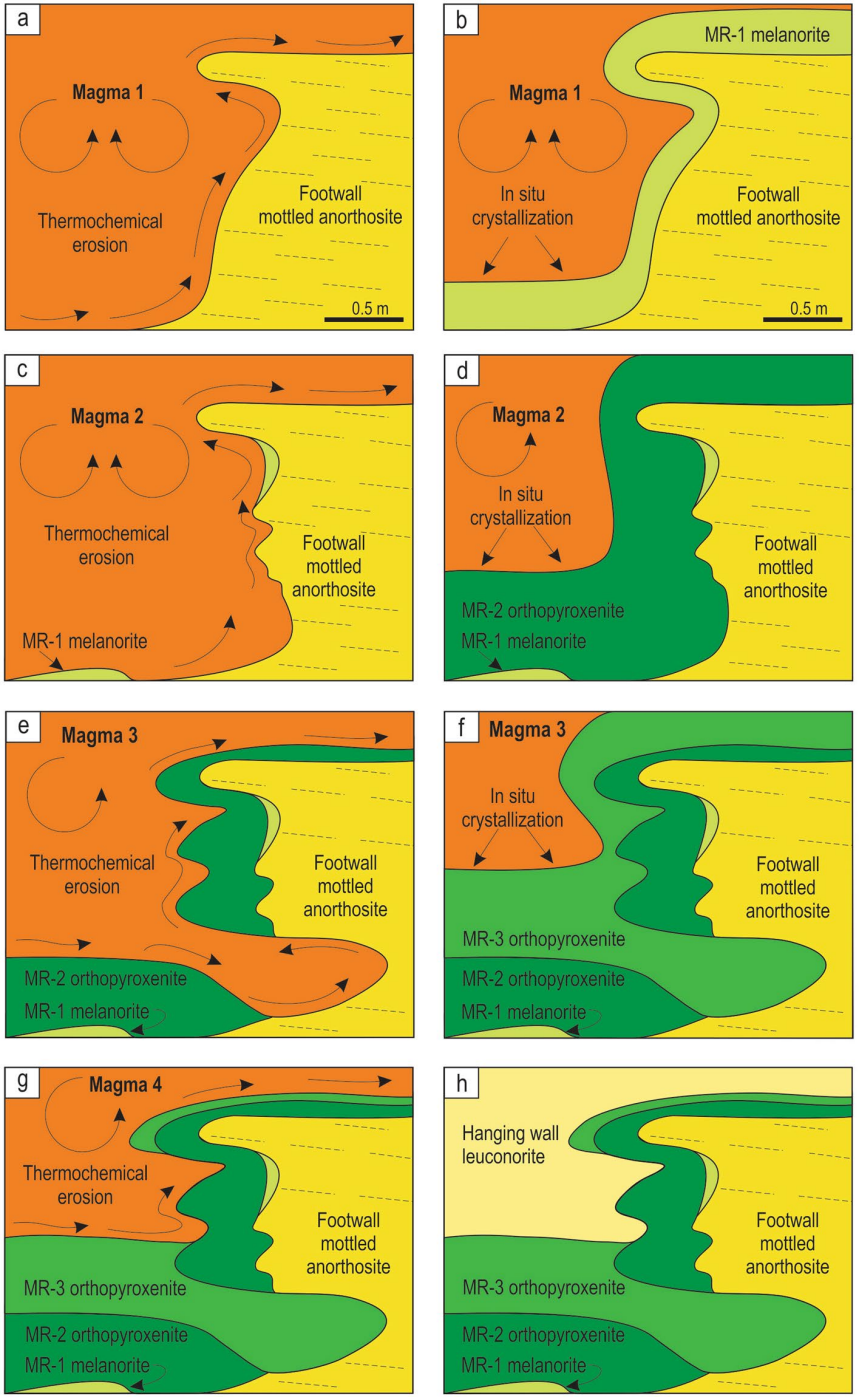
A physical model for the origin of the MR package of the Bushveld Complex through heterogeneous nucleation and *in situ* growth of crystals along the pothole margins. (**a**) First, a batch of dense, primitive and superheated magma entered the chamber and spread across the floor of the chamber as a basal layer. The superheated magma caused thermochemical erosion of the mottled anorthosites forming the temporary floor of the chamber, developing a pothole with subvertical to overhanging topology. (**b**) This was followed, upon magma cooling, by *in situ* growth of MR-1 melanorite with disseminated sulphides along the irregular erosional surface of the pothole. (**c**) A second batch of magma entered the chamber and spread across the floor of the chamber as a basal layer. The superheated nature of this magma resulted in thermochemical erosion of the earlier-deposited MR-1 melanorite. (**d**) The erosion was followed, on cooling, by *in situ* growth of the MR-2 orthopyroxenite with associated sulphides along the irregular erosional surface of the pothole. (**e**) A third batch of magma entered the chamber and spread across the floor of the chamber as a basal layer. The superheated nature of the magma resulted in thermochemical erosion of the earlier-deposited MR-2 orthopyroxenite and locally developed sheet-like cavities extending into the footwall rocks. The erosion was followed, upon magma cooling, by *in situ* growth of the sulphide-poor MR-3 orthopyroxenite on all irregularities of the erosional surface. Throughout the entire formation of the MR package (MR-1, MR-2 and MR-3 sublayers), droplets of sulphide melt scavenged PGE from a large volume of magma that was continuously brought towards the crystal-liquid interface by flowing/convecting magma. This allowed the sulphide melts to attain high concentrations of PGE. (**g**–**h**) The formation of the MR package was finally terminated by emplacement of a pulse of evolved noritic magma that, after some erosion of the earlier-deposited cumulates, crystallized the barren, hanging wall leuconorite.

The *in situ* growth of the MR in the studied exposure (Figs 3, 4) has been complicated by the fact that it is not the result of a single crystallization event, but rather forms through a series of erosional and depositional events. This is evident from the three compositionally distinct sublayers of the MR that have discordant relationships with each other (Fig. 4). We therefore explain the complex relationships in this exposure (Fig. 8) through multiple influxes of new dense, primitive and likely superheated magmas of varying compositions that spread laterally along the floor of the chamber as basal flows. The magma may have become superheated during its rapid ascent from depth, due to the difference in slope between the adiabatic gradient and the liquidus temperature. The superheating can reach a few tens of degrees if magma ascends from a storage region located at the depth corresponding to a pressure of about 10 kbar^62^. Such magmas are capable of extensive thermochemical erosion of both footwall anorthosites and melanorites/orthopyroxenites crystallized from the preceding magma pulses^24,25,27^ (Fig. 8a,c,e,g). Upon subsequent cooling, the magma pulses crystallized either melanorite or orthopyroxenite with or without associated sulphides (Fig. 8b,d,f). All phases, including droplets of an immiscible sulphide melt, form directly on the magma-cumulate interface because heterogeneous nucleation on pre-existing crystals is a kinetically favorable process^41,63^. This resulted in the MR sublayers draping all topographic irregularities of the chamber floor, including where it was steeply inclined or even entirely overturned. The scavenging of PGE by *in situ* forming sulphides was remarkably efficient due to extremely high partition of PGE into sulphide melt^64,65^ and a very low rate of crystal accumulation compared to that of magma convection in the chamber^33,34^. The *in situ* nucleation of an immiscible sulfide melt is somewhat surprising because the nucleation barrier for its formation is less than for a crystalline solid but occurrence of sulphides in overhangs clearly indicate that this was the case (Figs 3, 4, profile V). In addition, the higher PGE tenor along the subvertical to overhanging portions of the pothole (Fig. 5b) can be taken as yet another piece of evidence for *in situ* growth of sulphides: the rate of crystal accumulation on overhanging portions is expected to be lower than on planar ones allowing for the longer time for sulphides to sequester PGE from the convecting silicate melt. This happens because some crystals growing *in situ* on subvertical to overhanging portions are gravitationally unstable and may therefore be entrained downwards by convecting/flowing magma, resulting in the smaller thicknesses of the MR package along the margins and greater thicknesses at the base of potholes (Figs 3, 7a). Finally, the formation of the MR package was terminated by the basal emplacement of relatively evolved magma that, after some erosion of the MR package (Fig. 8g), crystallized into the barren, hanging wall leuconorite (Fig. 8h).

Our study has a direct implication for one fundamental question of modern petrology and volcanology. Historically, the concept of crystal settling was a major paradigm for the development of magma chambers^28–30^. It implies that crystals nucleate and grow throughout the entire volume of the chamber and then, due to gravitational forces, settle towards it base, causing the resident magma to differentiate^28–38^. The alternative idea considers magma chambers as nearly crystal-free and implies that all crystals nucleate and grow directly at the cooling margins of the chamber, with the evolved liquid from growing crystals being swept away and mixed with the overlying melt to cause its differentiation^40–52^. A large body of factual, experimental and theoretical observations have accumulated over decades on both sides of this controversy, but no sign of reaching a consensus is yet apparent^31,50,66,67^. Our discovery of cumulate layers developing along the overturned portions of the Bushveld chamber floor (Figs 2–4, 7) can be considered as arguably the strongest field-based evidence for *in situ* crystallization along cooling margins being a major factor in the development of magma chambers. This also suggests that, at least, within the Bushveld Complex, the evolution of the resident magma may have occurred via removal of the evolved liquid from *in situ* growth sites through compositional convection and mixing with the overlying melt. This discovery, taken together with a number of other textural and chemical features of layered intrusions^40–48^, indicates that the suggestion ‘when in doubt, settle it out’^42^, should be laid to rest, with all due respect to the memory of the great petrological luminaries who introduced the crystal settling concept^28–30^. For a long time, this concept was instrumental to our interpretation of the accumulating data from magma chambers, but the process is unlikely to be petrologically significant, unless crystals are brought into the chamber as intratelluric phenocrysts within replenishing magmas and are therefore freely available for gravity settling^35,37,39,49^. The analysis would be not complete without mentioning the recently advanced ‘mushy’ paradigm that denies the existence of large and long-lived magma chambers on Earth^68–70^. This paradigm invokes a transcrustal crystal-rich, liquid-poor mush that, in response to tectonically-induced deformation, occasionally produces small and ephemeral lenses of melt, which rapidly accumulate together to erupt via volcanoes as lavas on the Earth’s surface. We cannot expand our discussion to this novel paradigm because, in its current form, it mostly deals with felsic to intermediate magma chambers and it remains to be seen how it will be extended to those of basaltic to picritic composition. It is obvious, however, that this new emerging paradigm cannot be regarded as providing a unified view on igneous processes^70^ until it explains the existence of the Bushveld Complex – the largest and apparently long-lived basaltic magma chamber on Earth^17,30^ that hosts giant mineral deposits, including the Merensky Reef (Fig. 1b).

## Methods

### Geochemistry

One representative exposure of an overhanging MR package from a pothole in the Lonmin Platinum Karee mine, Western Bushveld was selected for detailed sampling. Bulk chemical analyses were obtained from five vertical and horizontal sections across the MR package in this exposure (Fig. 3). Rectangular slices 3–4 cm to 8–10 cm in size (100–300 g) covering the entire height of the MR were cut using a diamond saw. Major and trace elements were analysed by Genalysis Intertek Laboratory Services in Australia. Major elements were determined through X-ray fluorescence analyses (XRF) of compressed powder pellets. Calibrations used the international rock standard SARM8 as well as in-house controls. Agreement with recommended values was better than 0.6% for Cr_2_O_3_, Fe_2_O_3_, MgO, Al_2_O_3_ and better than for 1–6% for all other major elements. Trace elements were determined through inductively-coupled plasma mass spectrometry and atomic emission spectrometry (ICP-MS/ICP-AES) with four acid digests and nickel sulphide collection fire assay for PGE. Each ICP-MS analysis was accompanied by control standards GTS-2a, AMIS0167, and AMIS0013 and selected samples were reanalyzed to check anomalous results. For all elements the relative standard deviations were less than 10%. A complete list of analyses from the overhanging MR is available online within the electronic Supplementary Table 1. Petrography of the studied rocks is described in the Extended Data (see also Extended Data Fig. 1).

### XRF imaging

Microbeam scanning X-ray fluorescence analysis was conducted on a polished rock slab using the Bruker Tornado™ desktop scanner equipped with a rhodium target X-ray tube operating at 50 kV and 500 nA without filters and an XFlash® silicon drift X-ray detector. Analyses were quantified using the Bruker ESPRIT software, which provides standardless semi-quantitative analyses for elements heavier than Na, with estimated detection limits for the given operating conditions and dwell times of approximately 1000–2000 ppm for first-row transition metals. Maps were created using a 25–40 μm spot size on a 40 μm raster with dwell times of 10–20 ms per pixel. Maps are represented as un-quantified background-corrected peak height data for Kα peaks for each element.

## Supplementary information

Supplementary Information

Supplementary Dataset

## Data Availability

The authors declare that all relevant data are available within the article and its Supplementary Information files.
