# Supplementary material for: Merensky-type platinum deposits and a reappraisal of magma chamber paradigms
Source: Sci Rep. 2019 Jun 19;9:8807. doi: 10.1038/s41598-019-45288-8 (PMC6584556; doi:10.1038/s41598-019-45288-8)
Supplement: Supplementary file 1 — Supplementary Information [file 41598_2019_45288_MOESM1_ESM.pdf]

Supplementary Information:

Merensky-type platinum deposits and a reappraisal of magma chamber paradigms

Sofya Chistyakova[1]*, Rais Latypov[1], Emma J. Hunt[1] & Stephen Barnes[2]

Petrography:

The studied overhanging MR package consists of two major rock types (Figs. 4-5): melanorite (MR-1) and orthopyroxenite (MR-2 and MR-3). The package is sandwiched between the FW mottled anorthosite and HW leuconorite. The petrographical differences between the three sublayers of the MR package and their host rocks are highlighted below (Fig. 1).

The FW mottled anorthosite is characterized by an adcumulate texture with 85-95% cumulus plagioclase and 5-15% oikocrysts of orthopyroxene and clinopyroxene. The plagioclase crystals forming the bulk of the rock are euhedral and range in size from 0.3 to 2.5 mm. The rock typically displays a foliation fabric defined by a shape preferred orientation of the plagioclase crystals, with no evidence of any lineations. Orthopyroxene and clinopyroxene are interstitial and form oikocrystic grains up to 3-5 cm in size. The oikocrysts host numerous euhedral plagioclase crystals, which are slightly smaller than those outside them (Fig. 1a).

The HW leuconorite is medium-grained and composed of 65-80% cumulus plagioclase and 15-30% orthopyroxene with 5% interstitial clinopyroxene, 0.5% sulphides and secondary biotite and/or amphibole. The plagioclase crystals are subhedral to euhedral and range in size from 0.5 to 3.0 mm. The rock typically displays a foliation fabric defined by orientation of the plagioclase crystals, with no lineations. The orthopyroxene crystals are subhedral, range in size from 0.5 to 4.0 mm and commonly display clinopyroxene exsolution lamellae. Locally orthopyroxenes form oikocrysts enclosing plagioclase crystals. Clinopyroxene is always interstitial. Disseminated sulphides occur throughout the leuconorite. They typically occur interstitially between plagioclase crystals (Fig. 1b).

The MR-1 melanorite is marked by a higher modal content of orthopyroxene compared to the HW leuconorite. The melanorite is coarser-grained (up to 5 mm) and is composed of 50–55% plagioclase, 40–45% orthopyroxene, 5% clinopyroxene and 0.5% sulphides with secondary biotite and/or amphibole. Unlike leuconorite there is no evidence of any foliation fabric. The plagioclase crystals are subhedral to euhedral and range in size from 0.5 to 4.0 mm. The orthopyroxene crystals are subhedral, range in size from 1.0 to 5.0 mm and commonly display clinopyroxene exsolution lamellae. Locally orthopyroxene forms oikocrysts that enclose plagioclase crystals. Clinopyroxene is always interstitial. Disseminated sulphides occur interstitially between plagioclase and orthopyroxene crystals (Fig. 1c).

The orthopyroxenite (MR-2 and MR-3) is typically composed of 60-75% orthopyroxene, 15-30% plagioclase, 5-10% clinopyroxene and 0.5-5% sulphides with minor secondary biotite and/or amphibole. The main difference between MR-2 and MR-3 is in the amount of sulphides: about 5% in MR-2 and less than 1% in MR-3 (Extended Data Fig. 1d). Orthopyroxene is subhedral to euhedral and ranges in size from 1.0 to 7 mm in MR-2 orthopyroxenite and from 0.5 to 5 mm in MR-3 orthopyroxenite (Supplementary Information Fig. 1e, f). MR-2 orthopyroxenite is thus slightly coarser-grained than MR-3 orthopyroxenite. Orthopyroxenes typically show undulose extinction and commonly contain lenticular exsolution lamellae of clinopyroxene. In both orthopyroxenites (MR-2 and MR-3) plagioclase crystals most often have a subhedral to euhedral form and range in size from 1.0 to 4.0 mm; however, large interstitial crystals up to 4 mm also occur. Clinopyroxene is usually interstitial and forms oikocrysts up 5 mm, which may enclose small orthopyroxene and plagioclase crystals. Sulphide minerals are anhedral and are typically present as inclusions within, or interstitial to, orthopyroxene and plagioclase crystals, in close association with biotite grains.

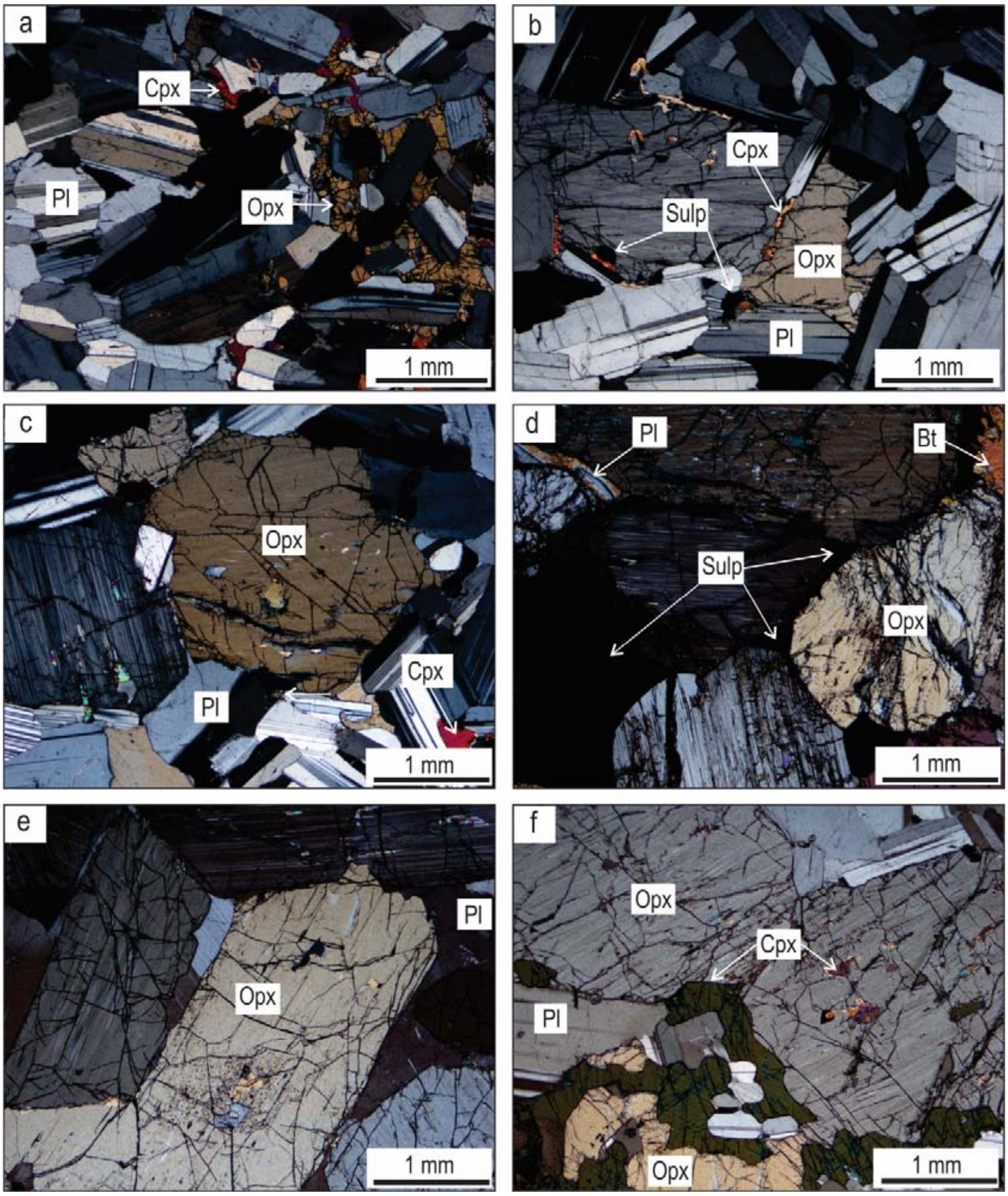

Figure 1. Photomicrographs of the overhanging MR and host rocks from the studied exposure at Shaft 3 of the Lonmin Platinum mine,Western Bushveld Complex (all photographed under crossed polarized light and are shown with the correct way up). **b** HW leuconorite with subhedral Opx crystals and disseminated sulphides (Sample III-1/2). **c** MR-1 melanorite with subhedral Opx grains and interstitial Cpx (Sample III-3-2). **d** MR-2 orthopyroxenite with sulphides filling in the interstices between cumulus orthopyroxene (Sample III-1/1) **e** MR-2 orthopyroxenite with large crystals of cumulus orthopyroxene and interstitial plagioclase (Sample I-8/1). **f** MR-3 orthopyroxenite with cumulus orthopyroxene and interstitial plagioclase and clinopyroxene (Sample II-3/3). Abbreviations: Pl - plagioclase, Opx - orthopyroxene, Cpx - clinopyroxene, Sulp - sulphides, Bt - biotite.
